# Structure of the malaria vaccine candidate Pfs48/45 and its recognition by transmission blocking antibodies

**DOI:** 10.1038/s41467-022-33379-6

**Published:** 2022-09-24

**Authors:** Kuang-Ting Ko, Frank Lennartz, David Mekhaiel, Bora Guloglu, Arianna Marini, Danielle J. Deuker, Carole A. Long, Matthijs M. Jore, Kazutoyo Miura, Sumi Biswas, Matthew K. Higgins

**Affiliations:** 1 Department of Biochemistry, South Parks Road, University of Oxford, Oxford, OX1 3QU UK; 2 Kavli Institute for Nanoscience Discovery, Dorothy Crowfoot Hodgkin Building, University of Oxford, South Parks Rd, Oxford, OX1 3QU UK; 3 Jenner Institute, University of Oxford, Old Road Campus Research Building, Roosevelt Drive, Oxford, OX3 7DQ UK; 4 Malaria Immunology Section, Laboratory of Malaria and Vector Research, National Institute of Allergy and Infectious Diseases, NIH, Rockville, MD USA; 5 Department of Medical Microbiology, Radboud University Medical Centre, Nijmegen, The Netherlands

**Keywords:** Vaccines, Parasitology, Malaria

## Abstract

An effective malaria vaccine remains a global health priority and vaccine immunogens which prevent transmission of the parasite will have important roles in multi-component vaccines. One of the most promising candidates for inclusion in a transmission-blocking malaria vaccine is the gamete surface protein Pfs48/45, which is essential for development of the parasite in the mosquito midgut. Indeed, antibodies which bind Pfs48/45 can prevent transmission if ingested with the parasite as part of the mosquito bloodmeal. Here we present the structure of full-length Pfs48/45, showing its three domains to form a dynamic, planar, triangular arrangement. We reveal where transmission-blocking and non-blocking antibodies bind on Pfs48/45. Finally, we demonstrate that antibodies which bind across this molecule can be transmission-blocking. These studies will guide the development of future Pfs48/45-based vaccine immunogens.

## Abstract (web summary)

Pfs48/45, a surface protein of *Plasmodium falciparum*, is a promising anti-malarial vaccine candidate whose structure is not entirely resolved. Here, the authors present the structure of the full-length molecule, and characterise the binding and activity of transmission blocking antibodies.

## Introduction

The recent approval of RTS,S as the first vaccine to prevent malaria caused by *Plasmodium falciparum* is an important step in efforts to end the devastating effects of this ancient disease^1^, with promising results also recently released for the related R21 vaccine^2^. However, with large-scale trials of RTS,S indicating that it is around 30% effective at preventing severe disease^3^, the quest for a more effective vaccine continues. The *Plasmodium* life cycle is complex and there are multiple stages at which such a vaccine could act^4^, including stopping fusion of the male and female gametes of the parasite within the mosquito midgut. If the bloodmeal of a mosquito contains antibodies which prevent gamete fusion, then these can stop completion of the parasite life cycle and can block transmission. Vaccine immunogens which induce the production of such transmission-blocking antibodies will therefore be important components of multi-stage-targeting malaria vaccines^5^.

One of the most promising transmission-blocking vaccine candidates is the gamete surface protein, Pfs48/45. This is essential for gamete fusion, as male *Plasmodium berghei* gametes that lack the homologous protein, Pbs48/45, are unable to penetrate female gametes to form zygotes^6^. In addition, antibodies induced by immunising animals with Pfs48/45 block the sexual development of the parasite within infected mosquitos^7–12^. Pfs48/45 is expressed on gametocytes found in human blood and is therefore exposed to the human immune system. Indeed, antibodies that target Pfs48/45 are also found in sera from individuals from malaria-endemic regions and the presence of such antibodies correlates with the transmission-blocking activity of these sera^13–17^. This means that individuals immunized with Pfs48/45 could also experience immune boosting through natural low-level infection. Also encouraging is the low sequence diversity of Pfs48/45 across strains of *Plasmodium falciparum*^12,16,18–20^. These factors combine to suggest that a vaccine immunogen based on Pfs48/45 will target a conserved and essential feature of the parasite life cycle and prevent further transmission from a malaria-infected individual.

As full-length Pfs48/45 has proved challenging to produce, vaccine immunogen design has so far been guided by an understanding of the molecular architecture of Pfs48/45 and by the identification of epitopes for potent monoclonal antibodies^5,21^. Pfs48/45 is formed from three domains, with N- and C-terminal 6-cys domains joined by a central 4-cys domain, all linked to the gamete membrane through a C-terminal GPI anchor^22–24^. While these domain boundaries are well understood, how they come together to form full-length Pfs48/45 is still not known. Monoclonal antibodies have been generated against Pfs48/45, have been classified into different competition groups, and have been assessed for their transmission-blocking activity^8,12,25–28^. The most potent of these antibodies, 32F3^8^ and 85RF45.1^25^ bind to the C-terminal domain and the structure of the C-terminal domain bound to the Fab fragment of 85RF45.1 has been determined^12,29^. This domain has therefore been the focus of most vaccine immunogen design approaches targeting Pfs48/45, primarily administered as part of fusion proteins, such as that with GLURP^21^. However, other transmission-blocking antibodies have been identified which target the N-terminal and central domains of Pfs48/45^12,25,28^. Here, we therefore present the structure of the full Pfs48/45 ectodomain, show how antibodies recognise different regions of Pfs48/45 and demonstrated that a significant fraction of the transmission-blocking activity of sera targeting Pfs48/45 is due to antibodies which target its N-terminal and central domains.

## Results

### A structural comparison of two antibodies targeting domain 3 of Pfs48/45

**Fig. 1:**
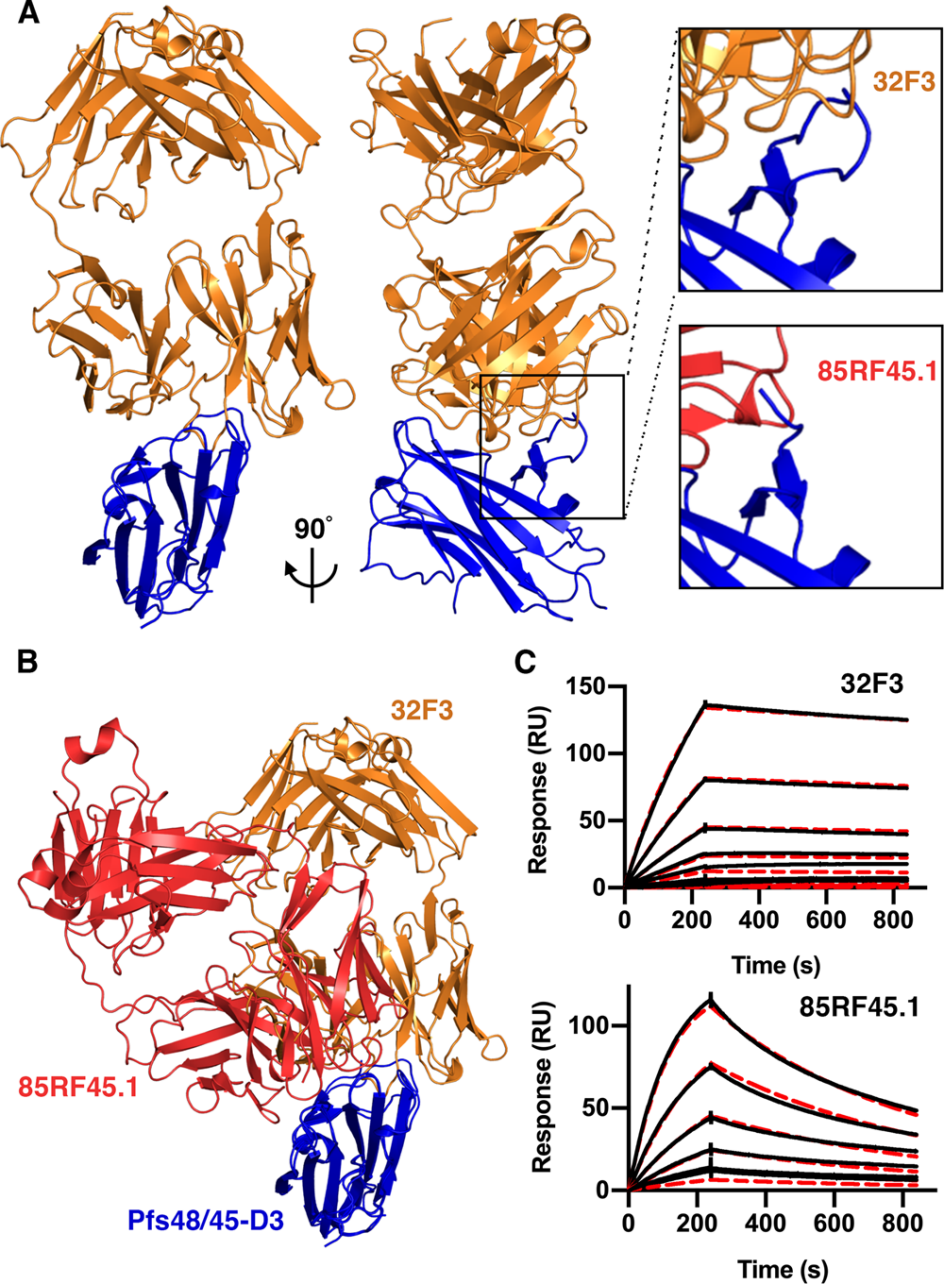
Comparison of the epitopes of transmission-blocking antibodies 85RF45.1 and 32F3. **A** The structure of the C-terminal domain of Pfs48/45 (Pfs48/45-D3; blue) bound to antibody 32F3 (orange). The upper right inset shows a close-up of a Pfs48/45 loop which becomes ordered on 32F3 binding. The lower inset shows the equivalent view of the complex of Pfs48/45-D3 (blue) bound to 85RF45.1 (red). **B** An alignment of the structures of 32F3 (orange) and 85RF45.1 (red) bound to Pfs48/45-D3 (blue). **C** Surface plasmon resonance analysis of the binding of Pfs48/45-D3 to immobilised 32F3 and 85RF45.1. In both cases, the black lines show the responses due to a two-fold dilution series with a top concentration of 7.8 nM for 85RF45.1 and 125 nM for 32F3. The red dashed lines show fitting to a 1-to-1 binding model.

The two antibodies which have the highest transmission-blocking activity, 32F3 and 85RF45.1, both bind to the C-terminal domain of Pfs48/45^8,12^. However, these two antibodies show substantial differences in transmission-blocking activity, with 85RF45.1 completely preventing transmission at 14 μg/ml, while 32F3 is only ~40% effective at 70 μg/ml^12^. To rationalise these differences, and to guide future vaccine design, we determined the structure of the C-terminal 6-cys domain of Pfs48/45 bound to 32F3 (Fig. 1A, Supplementary Table 1). We prepared Fab fragments from 32F3, combined these with the Pfs48/45 C-terminal domain and conducted crystallisation trials. Crystals formed and a complete dataset was collected to 1.9 Å resolution, allowing structure determination by molecular replacement.

The structure of the C-terminal domain of Pfs48/45 was largely unchanged in conformation from that observed in complex with 85RF45.1, with a root mean square deviation of 0.66 Å (Fig. 1A, B)^12,29^. The most substantial change was in the loop comprising residues 357–369. This loop is disordered when bound to antibody 85RF45.1 but becomes ordered through interactions with 32F3 (Fig. 1A). While antibodies 32F3 and 85RF45.1 bind to overlapping epitopes, many of the contacts are not shared and the Fabs approach Pfs48/45 at different angles.

We also determined the binding kinetics for both 85RF45.1 and 32F3 to Pfs48/45, using surface plasmon resonance measurements (Fig. 1C, Supplementary Fig. 1). We first captured 85RF45.1 and 32F3 on different flow paths of a chip coated with protein A/G and flowed two-fold dilution series of Pfs48/45 over these antibody-coupled surfaces. Both antibodies bound to Pfs48/45 with similar affinities in the low nanomolar range (2.5 nM for 85RF45.1 and 5.2 nM for 32F3). However, binding kinetics differed, with 85RF45.1 showing faster association- and dissociation-rates, while 32F3 binds more slowly but forms a more stable complex. It seems likely that the slower binding kinetics of 32F3 may be due to the need for loop 357–369 to become ordered on binding, while antibody 85RF45.1 has an epitope which is unchanged in structure on antibody binding, allowing faster binding kinetics.

### Determining the structure of full-length Pfs48/45 by combining crystallography with an AlphaFold2 model

We next aimed to determine the structure of the complete Pfs48/45 molecule, allowing us to reveal the architecture of this three-domain protein and the locations of antibody epitopes outside the C-terminal domain. Insect cell expression systems were available to produce full-length Pfs48/45 ectodomain, or to generate a protein containing the central and C-terminal domains, Pfs48/45-D2 + 3 [11]. Both were expressed without the GPI-anchor modification site and were purified and mixed in different combinations with one or more Fab fragments, selected from a set of antibodies which bind to different domains of Pfs48/45 (85RF45.1, 85RF45.3, 85RF45.5^25^, 32F3^8^, 10D8, 9D1, 7A6^12^). Three of these complexes generated crystals: full-length Pfs48/45 bound to 10D8; full-length Pfs48/45 bound to both 10D8 and 85RF45.1; and Pfs48/45-D2 + 3 bound to both 10D8 and 32F3. Datasets were collected to 4.2 Å, 3.72 Å and 3.69 Å, respectively.

**Fig. 2:**
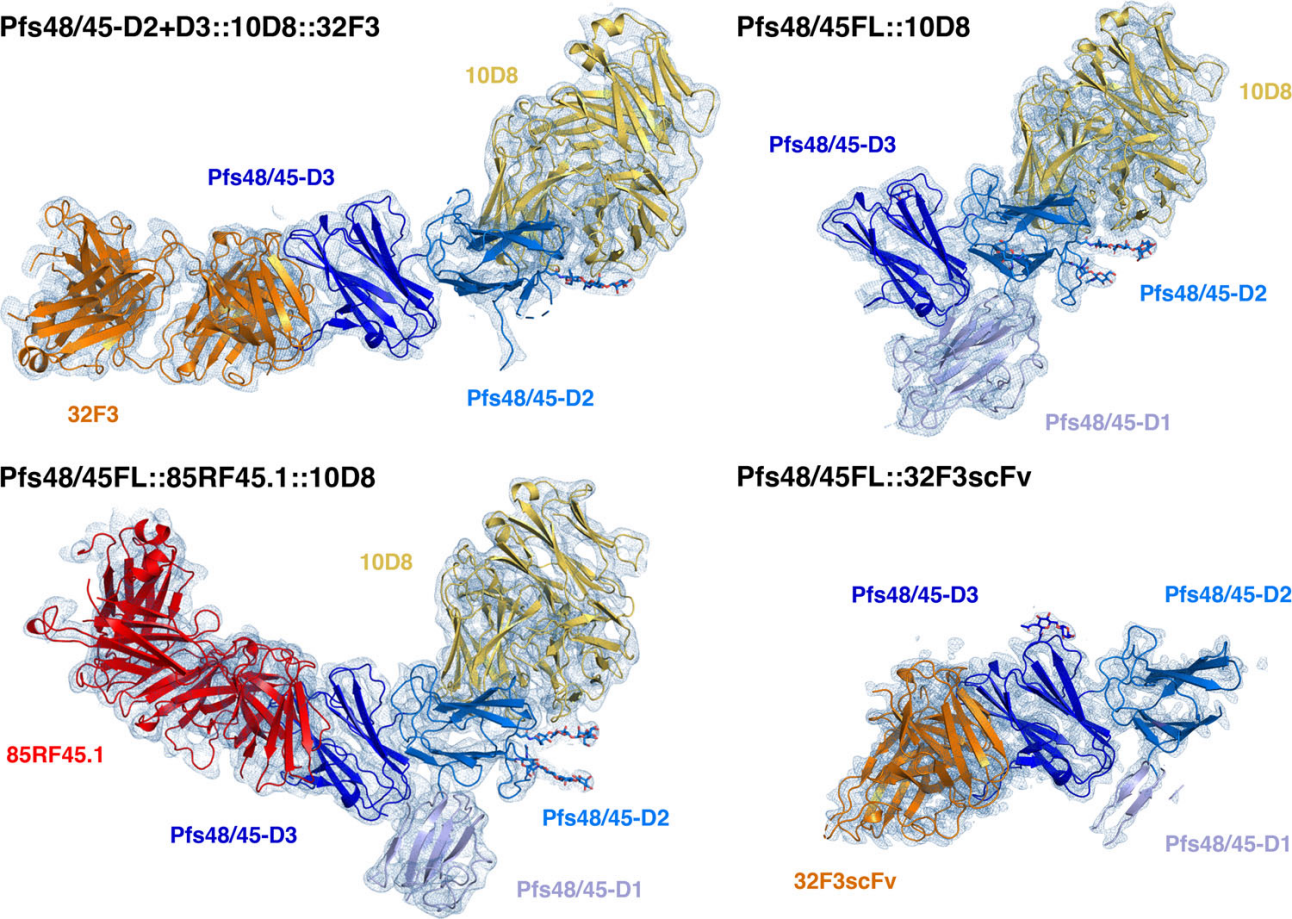
Structures of Pfs48/45 bound to antibodies 85RF45.1, 32F3 and 10D8. Structures of Pfs48/45 bound to different combinations of Fab fragments or scFvs. Pfs48/45 is shown with the three domains in different shades of blue. The N-terminal domain is light blue, the central domain is mid-blue and the C-terminal domain is dark blue. 10D8 is yellow, 85RF45.1 is red and 32F3 and its scFv are orange. Each figure also shows the electron density in pale blue, shown at a contour threshold of 1.0.

We attempted to determine the structures of these complexes by molecular replacement, using the structures of the C-terminal domain bound to either 32F3 or 85RF45.1 Fab as search models. In each case, the resultant electron density maps were sufficiently detailed to allow us to build a model for the central domain of Pfs48/45. This domain contains the epitope for 10D8, also allowing us to build a model for the 10D8 Fab (Fig. 2, Supplementary Tables 1 and 2). However, while electron density could be observed for the N-terminal domain of Pfs48/45, this region was not sufficiently well resolved to allow a model to be built.

The hinge between the variable and constant domains of Fabs is flexible. In case this flexibility reduced the degree of order within the crystals, we also generated scFv constructs for 85RF45.1, 32F3 and 10D8. These were purified, mixed with full-length Pfs48/45 and complexes were subjected to crystallisation trials. Crystals formed for the complex containing 32F3 scFv and a dataset was collected to 2.13 Å resolution, allowing structure determination by molecular replacement. This higher resolution data allowed us to build an improved model for the central domain of Pfs48/45 (Fig. 2). However, the density for the N-terminal domain was still too poorly resolved to allow building of a complete molecular model of this domain. Analysis of packing within the different crystals suggested that, in each case, the N-terminal domain lies within a substantial cavity within the crystal lattice, and the lack of packing against other regions of Pfs48/45 contributed to disorder of this domain, even within a well-ordered lattice.

Recent improvements in protein structure prediction from AlphaFold2^30^ provided a solution, allowing us to interpret the electron density maps for the N-terminal domain. AlphaFold2 correctly predicted the architecture of the central and C-terminal domains (with root mean square deviation of 1.26 Å for the central domain and 0.43 Å for the core 753/1044 atoms of the C-terminal domain), albeit with differences in loop structure (accession code Q8I6T1 at alphafold.ebi.ac.uk) (Supplementary Fig. 2). We therefore compared the AlphaFold2 model for the N-terminal domain with the electron density for this domain within the three different maps. The most complete electron density for this region of Pfs48/45 was obtained from crystals of full-length Pfs48/45 bound to 10D8. The AlphaFold2 model was therefore docked as single rigid body into this electron density and was rebuilt to fit the density. This generated a model for 122 residues of the 150 residue-long N-terminal domain, with a root mean square deviation of 1.63 Å from the AlphaFold2 model. The N-terminus and loops 62–68 and 163–168 were unresolved. This model was then used to guide building of the observed portions of the N-terminal domain in full-length Pfs48/45 bound to 10D8 and 85RF45.1 (95 residues were built) and Pfs48/45-D2 + 3 bound to both 10D8 and 32F3 (20 residues were built). Through this approach, we generated the first molecular models of full-length Pfs48/45 derived from experimental crystallographic data, with an AlphaFold2 model used to guide building of the N-terminal domain (Fig. 2, Supplementary Tables 1–3).

### Pfs48/45 forms a dynamic three-domain disc-shaped architecture

**Fig. 3:**
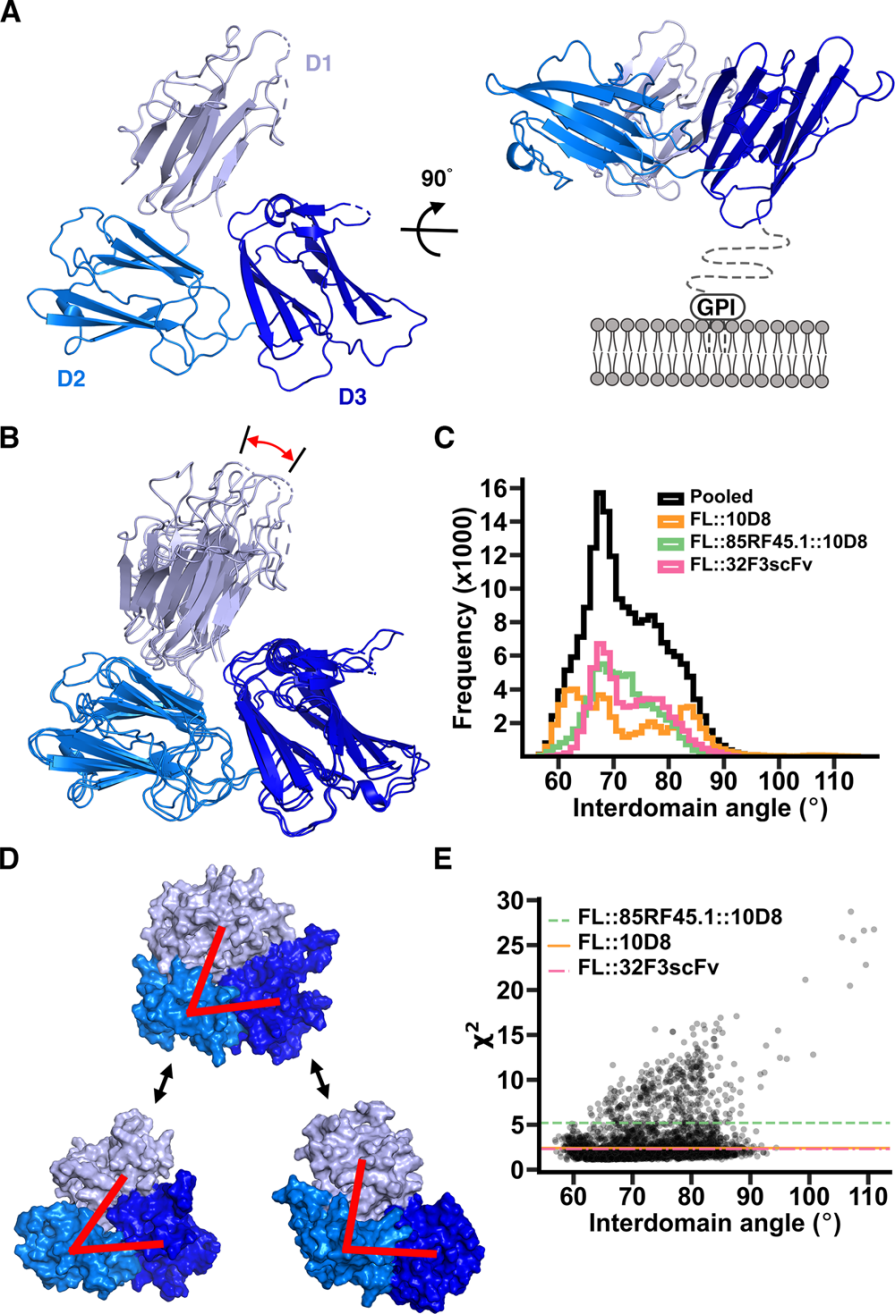
Structure and dynamics of Pfs48/45. **A** The structure of Pfs48/45 taken from that of full-length Pfs48/45 bound to 10D8, viewed from two different directions. The N-terminal (D1), central (D2) and C-terminal domains (D3) are light, medium and dark blue. The right-hand panel also indicates the membrane and the 20 residue long linker not included in our constructs. **B** Comparison of the three full-length Pfs48/45 structures. Three structures were constructed by taking the models of Pfs48/45 from the three different crystal structures of Pfs48/45 bound to different antibody combinations and aligning the structure of the N-terminal domain onto the fragments of the domain built into the electron density. These composite structures have been aligned based on the central and C-terminal domains, showing the motion of the N-terminal domain, highlighted by the red arrow. **C** Histogram showing the observed interdomain angles in full-length Pfs48/45 during atomistic molecular dynamics simulations. To define these angles two lines were drawn, linking the centres of mass of the N-terminal and central domains and those of the central and C-terminal domains. The angle shown is that between these lines. The three coloured histograms refer to outcomes of three independent simulations which started with the models in panel (**B**) with histograms being constructed by pooling five replicates for each starting structure. The black histogram is the pooled distribution of all replicates in all three starting models. **D** The average angle observed in the simulations from (**C**). is shown top-centre with the most closed observed model bottom-left and the most open model bottom-right. Red lines are as described in **C**. **E** shows the fitting of models from the molecular dynamic simulations to data from small angle X-ray scattering. Each point represents a different model and the *χ*^2^ gives the quality of fit to the scattering curve. Horizontal lines show the *χ*^2^ for the fit corresponding to interdomain angles found in the three crystal structures, as seen in **B**.

We next used our structures, together with molecular dynamics simulations, to understand the organisation of Pfs48/45. Our three structures of Pfs48/45 all reveal a flattened, disc-like architecture (Fig. 3A), with a 20 residue long linker before the GPI anchor. This membrane-attachment site emerges from a flat surface of the molecule, suggesting that the disc may lie, on average, parallel to the membrane, with all three domains equally exposed at the gametocyte surface.

The availability of three structures of Pfs48/45 allowed us to assess its degree of flexibility within crystals. We docked the most complete model of the N-terminal domain, from the structure of full-length Pfs48/45 bound to 10D8, onto the fragments of the domain observed in the two other crystal forms and the three resultant models for the full Pfs48/45 ectodomain were aligned (Fig. 3B). While the relative positions of the central and C-terminal domains were largely unchanged across these molecules, the position of the N-terminal domain changed, due to flexibility in the linker joining the N-terminal and central domains. Through these movements, the separation between the N- and C-terminal domains varied.

To further assess the degree of motion within Pfs48/45, we used molecular dynamics simulations. Three separate simulations were run, with our three independent structures of Pfs48/45, each modified to include the aligned N-terminal domain, used as three distinct starting points. In each case, we simulated the system for 500 ns with 5 repeats. Analysis of these simulations revealed motion of the N-terminal domain in excess of that seen in the three crystal structures, leading to further separation of the N- and C-terminal domains. The two peaks seen in some of the individual simulations were not observed in a pooled simulation, suggesting that each individual simulation had not converged, with pooled simulations reaching a more representative endpoint. We plotted two lines though the structure; one linking the centres of mass of the N-terminal and central domains and one linking the centres of mass of the central and C-terminal domains. The angle between these lines was used as a measure of the degree of opening of the complex. This varied from 56.4° to 114.9°, with an average of 72.8° and a standard deviation of 7.3° (Fig. 3C, D, Supplementary Fig. 3). The major peak varied from 56.4° to 92.1°, although we also observed a single instance of extreme opening to 112.7° (Supplementary Fig. 3A). This compares with angles of 60.3°, 63.1° and 67.5° in the three crystal structures.

To compare the fit of our molecular dynamic simulations to experimental data, we subjected Pfs48/45 to small angle X-ray scattering (Supplementary Fig. 4). We then sampled the molecular dynamic simulation trajectories to generate a PDB file for every 50th frame and fitted each of these to the scattering data using FoxS^31^ thereby generating a *χ*^2^ which allows us to quantify the fit. For comparison, we also fit the input models used for simulations, with domain positions matching those found in the three crystals. We then plotted the *χ*^2^ against the interdomain angle (Fig. 3E). This analysis shows that no single angle fits most closely to the SAXS data, with a broad range of models, with interdomain angle 56° from 93°, all fitting with a lower *χ*^2^ than any of the crystal structures. Indeed 66% of the models derived from simulation fitted the SAXS scattering data more closely than the three crystal structures. This confirms that Pfs48/45 in solution is a dynamic molecule, sampling a wide range of interdomain angles, through movement of the N-terminal domain relative to the central and C-terminal domains. This flexibility may allow Pfs48/45 to adopt different conformations on binding to partners, such as Pfs230 and will also leave all three domains exposed to antibody recognition.

### Mapping transmission-reducing antibody epitopes to each domain of Pfs48/45

**Fig. 4:**
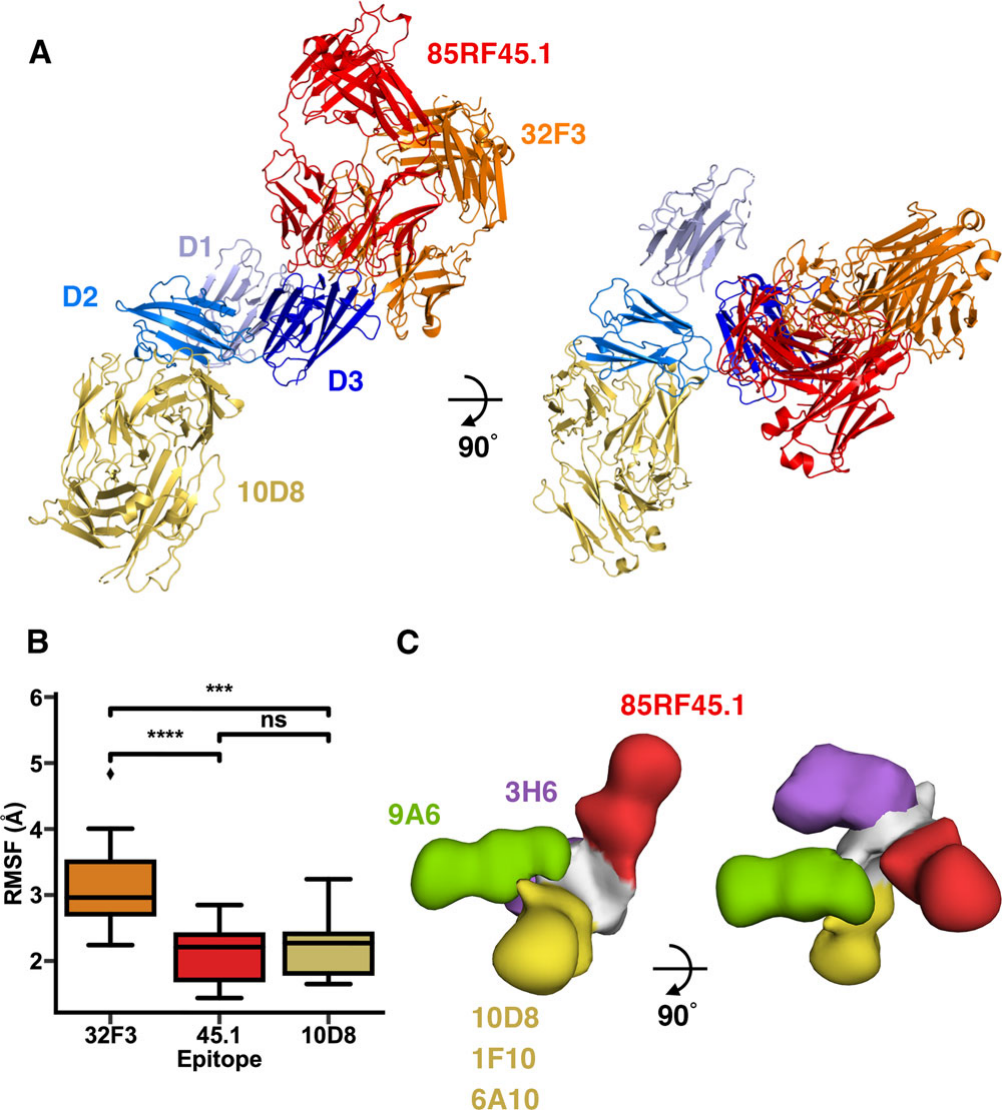
distribution of epitopes for transmission-blocking antibodies across Pfs48/45. **A** The structure of Pfs48/45 bound to 10D8 is used as a template, and combined with structures of the D3 domain of Pfs48/45 bound to either 85RF45.1 or 32F3 to generate a model, showing the relative locations of the three antibody epitopes. **B** A measure of the flexibility of the residues which form the epitopes for 32F3, 85RF45.1 and 10D8, as derived from molecular dynamics simulations. Each epitope was analysed in all 15 replicates and significance levels are based on a two-sided Mann–Whitney U tests where *** indicates *p* < 0.0005 and **** indicates *p* < 0.00005. The box bounds are interquartile ranges, and the lines within boxes are the median values. Whiskers extend to 1.5-fold the interquartile range. For 32F3 vs 85RF45.1 *p* = 0.00008; for 32F3 vs 10D8 *p* = 0.00031; for 10D8 vs 85RF45.1 *p* = 0.58974. **C** A composite model, derived from negative-stain electron microscopy structures. In each case, the complexes contained Pfs48/45 bound to 85RF45.1, together with either 9A6, 3H6, 10D8, 1F10 and 6A10. These were imaged by negative stain electron microscopy, and models were fitting into the resultant envelopes. These models were used to align the envelopes, allowing us to derive a composite model, showing the location for the five epitopes.

We next combined crystallography, electron microscopy and molecular dynamics simulations to assess how different monoclonal antibodies bind to Pfs48/45. In addition to visualising antibodies 32F3 and 85RF45.1 (Fig. 1), our crystal structures revealed the epitope for 10D8 (Fig. 2), an antibody with weak transmission-reducing potential which binds to the central domain of Pfs48/45^12^. We combined the structure of Pfs48/45 bound to 10D8 with those of the C-terminal domain bound to 32F3 and 85RF45.1 to generate a composite model, showing the structure of Pfs48/45 bound to these three antibodies (Fig. 4A).

To understand the degree of flexibility of these three epitopes we analysed the molecular dynamics simulations presented in Fig. 3, specifically assessing the motion of residues directly in contact with each of the three antibodies. This revealed that the epitope for 32F3 is significantly more flexible than those for 85RF45.1 and 10D8 (Fig. 4B), predominantly due to motion of the 357–369 loop, which becomes ordered on 32F3 binding due to direct interactions with the antibody (Supplementary Fig. 3). This indicates that the slower association-rate for 32F3, compared to the association rates for either 85RF45.1 or 10D8, is due to this flexibility and to the need for the epitope to adopt the correct structure on antibody binding. In contrast, off-rate is likely to be determined by the degree of complementarity of the epitope and paratope when forming an interaction. Indeed, 32F3 has the slowest off-rate (Supplementary Fig. 1), which correlates with a larger buried surface area for the epitope (1107 Å^2^ for 32F3, 875 Å^2^ for 85RF45.1 and 715 Å^2^ for 10D8) and a greater number of direct interactions (Supplementary Fig. 3)^12^.

Finally, we used negative stain electron microscopy to visualise the approximate location on Pfs48/45 of the epitopes of four more antibodies. 1F10 and 6A10 are in the same competition group on the central domain as 10D8 while 9A6 is in a different competition group, binding to the same domain^12^. In contrast, 3H6 binds to the N-terminal domain. While 1F10 and 6A10 show some transmission-reducing activity, 9A6 and 3H6 do not^12^. In each case, we assembled complexes containing Pfs48/45, 85RF45.1 Fab and one other antibody Fab, with 85RF45.1 included to provide a clear marker which could be used to align and position each additional antibody. We then used negative stain electron microscopy to image the complexes and single particle analysis to determine a low-resolution structure. The Pfs48/45:85RF45.1 complex and an additional Fab model were then docked into these structures, taking into account to which domain the additional Fab bound to determine the organisation of the complex and the approximate location of the Fab. These five models were then aligned on Pfs48/45 and assembled together to show the locations of the five antibodies (Fig. 4C, Supplementary Fig. 5, Supplementary Table 4). The locations of 10D8, 1F10 and 6A10, which are part of the same competition group, were superimposable. In contrast, 9A6 and 3H6 adopted different locations on Pfs48/45, with both protruding in approximately the same plane as that shared by the three domains of Pfs48/45.

These findings suggest that both accessibility and also unknown functional properties of Pfs48/45 contribute to the transmission-blocking efficacy of antibodies which target Pfs48/45. When attached to the membrane, through a C-terminal GPI anchor, we would expect the Pfs48/45 disc to be on average arranged horizontal to the membrane plane (Fig. 3A). In this orientation, the epitope for 85RF45.1 will be most exposed on the membrane surface with the 32F3 epitope exposed to a lesser degree and the 10D8 epitope least accessible. This correlates with the order of transmission-blocking activity of these three antibodies, with 85RF45.1 as most potent and 10D8 as least potent. However, the epitopes for 9A6 and 3H6 emerge in the plane of Pfs48/45 and we would expect them to be more regularly exposed on the membrane surface than the epitope for 10D8, and yet they are not transmission-blocking^12^. It is also notable that neither 3H6 or 9A6 stain gametocytes, suggesting these epitopes to be buried in that context^12^. A possible explanation for this is that the region of Pfs48/45 bound by these antibodies may be occluded by its binding partners, such as Pfs230. Further studies of Pfs48/45 function are required to determine whether this is the case.

### Antibodies targeting the N-terminal and central domains of Pfs48/45 substantially contribute to transmission-blocking activity of sera

The structure of Pfs48/45 indicates that each of its three domains will be exposed on the gametocyte surface (Fig. 3) and antibodies binding to each domain have been shown to stain gametocytes^12^. However, the most effective known transmission-blocking monoclonal antibodies bind to the C-terminal domain^8,25^. We therefore aimed to determine the degree to which antibodies against the N-terminal and central domains contribute to transmission-blocking activity, using a combination of immunisation and antibody depletion experiments.

**Fig. 5:**
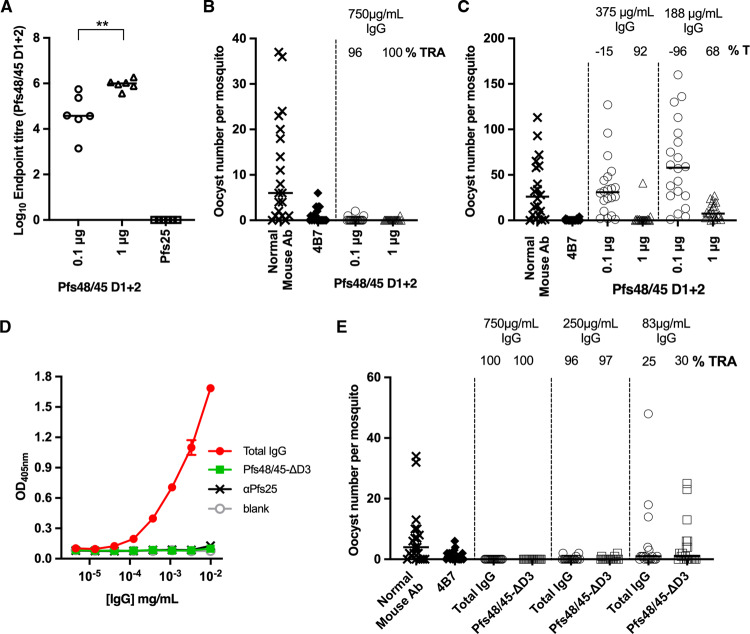
Transmission-blocking antibodies target the N-terminal and central domains of Pfs48/45. **A**–**C** Female CD1 mice (*n* = 6 mice) were immunised twice with either 0.1 μg or 1 μg of Pfs48/45-D1 + 2, or 1 μg of Pfs25. Sera was collected three weeks after the final dose for analysis. **A** IgG titres as measured by endpoint ELISA using Pfs48/45-D1 + 2 as the coating antigen. Each symbol is for the serum sample from an individual mouse; lines represent the median of each group. Mann–Whitney two-tailed test was performed to compare the two Pfs48/45-D1 + 2 groups (*p* = 0.0043). **B**, **C** Transmission-blocking efficacy of IgG induced by immunisation with Pfs48/45-D1 + 2. Total IgG was purified from the pooled serum of each group (3 weeks post-boost) and mixed with *P. falciparum* NF54 cultured gametocytes at 750 μg/mL (**B**) and in a separate experiment at 375 μg/mL and 83 μg/mL (**C**) and fed to *A. stephensi* mosquitoes (*n* = 20 mosquitoes per test group of pooled IgG in a single feed experiment). IgG from naive mice was used as a negative control (“normal mouse Ab”); the transmission-blocking anti-Pfs25 mAb 4B7 was used as a positive control at a concentration of 94 μg/mL. Data points represent the number of oocysts in individual mosquitoes 8 days post-feeding; horizontal lines show the arithmetic mean. **D**, **E** Female CD1 mice were immunised three times with 5 μg of Pfs48/45-FL. Three weeks after the final dose sera were collected and total IgG purified. Total IgG was depleted of Pfs48/45-D3-targeting IgG using a column coupled with Pfs48/45-D3, resulting in Pfs48/45-ΔD3. **D** Pfs48/45-D3 specific ELISA showing lack of recognition of Pfs48/45-D3 by Pfs48/45-ΔD3. Pfs25-specific IgG (αPfs25) was included as a negative control. Data presented as mean +/− standard deviation. **E** Transmission-blocking efficacy of total IgG from Pfs48/45-FL or Pfs48/45-ΔD3 fed to *A. stephensi* mosquitoes at concentrations of 750 μg/mL, 250 μg/mL and 83 μg/mL (*n* = 20 mosquitoes per test group of pooled IgG in a single feed experiment). IgG from naive mice was used as a negative control (“normal mouse Ab”); transmission-blocking anti-Pfs25 mAb 4B7 was used as a positive control. Data points represent the number of oocysts in individual mosquitoes; lines show the arithmetic mean (TRA, transmission reducing activity [% inhibition in mean oocyst count per mosquito]).

We first produced protein consisting of the N-terminal and central domains of Pfs48/45 (Pfs48/45-D1 + 2) and used this to immunise mice. IgG was purified from these mice and the titres of antibodies targeting Pfs48/45-D1 + 2 were determined by end-point ELISA (Fig. 5A). The transmission-blocking activity of these antibodies was then assessed using a standard membrane feeding assay. Antibodies were added to blood containing *Plasmodium falciparum* gametocytes, fed to mosquitos, and the number of ookinetes formed in mosquito midguts was counted. Total IgG, purified from mice immunised with either 0.1 μg or 1 μg of Pfs48/45-D1 + 2 was tested at a concentration of 750 μg/ml, causing almost complete inhibition (96 and 100% reductions resulting from 0.1 μg and 1 μg immunisations, respectively) in oocyte numbers (Fig. 5B). When tested at 375 and 188 μg/ml, the total IgG from the 1 μg group also showed significant inhibition of 92 and 68%, respectively (Fig. 5C). Therefore, protein immunogens containing just the N-terminal and central domains of Pfs48/45 are able to induce highly potent transmission-blocking antibodies.

We next used a depletion approach to assess the contribution that antibodies which target the N-terminal and central domains of Pfs48/45 make to the transmission-blocking activity of IgG from mice immunised with full-length Pfs48/45. Total IgG from mice immunised with 5 μg of full-length Pfs48/45 was tested at a concentration of 750 μg/ml and showed 100% transmission-blocking activity (Fig. 5D, E). This IgG was depleted of antibodies which bind to the C-terminal domain of Pfs48/45 using an affinity column and was tested for transmission-blocking activity (Fig. 5D, E). Depletion of C-terminal domain (D3) specific antibodies from purified IgG was confirmed by ELISA, using Pfs48/45-D3 as the coating antigen, with no signal observed in the depleted IgG compared to the original un-depleted IgG (Fig. 5D).

At all three total IgG concentrations tested, the sera depleted of C-terminal domain-targeting antibodies showed no significant difference to the sera prior to depletion (Fig. 5E). This shows that the majority of the antibodies with transmission-blocking activity, induced through immunisation with full-length Pfs48/45, bind to either the N-terminal or central domains.

## Discussion

The structure of the Pfs48/45 ectodomain reveals that its three domains are arranged as a disc. The location of the C-terminal GPI-anchor attachment site on one of its flatter surfaces will cause the disc to lie approximately parallel to the membrane plane, with all three domains accessible on the cell surface, albeit with the flexible linker of 20 residues between the ordered section allowing considerable movement. The presence of a flexible linker between the N-terminal and central domains also causes the disc to be dynamic, due to opening and closing of the gap between the N- and C-terminal domains. How this structure and dynamics relate to the function of Pfs48/45 is currently unclear. Pfs48/45 on gametocyte surfaces is part of a larger protein complex including other proteins essential for gamete fusion, including Pfs230^32^ and the PfCCp proteins^33,34^. The molecular architecture of this complex and the mechanistic roles of its components during gamete fusion, are currently not known. The structure of Pfs48/45 makes it tempting to speculate that the open, dynamic membrane-distal surface of the disc, as presented on gametocytes, acts as a platform that allows it to bind important interaction partners such as Pfs230 and the PfCCp proteins. To test this will require further study, both to determine how this complex is arranged and to understand which parts of Pfs48/45 are occluded by binding partners.

The structure of Pfs48/45 also has implications for our understanding of what makes an effective transmission-blocking antibody. A number of criteria might determine the potency of such an antibody, including the location of its epitope relative to functionally important sites on the protein, its binding kinetics and the accessibility of its epitope when presented on the parasite surface. Our lack of mechanistic insight into the role of Pfs48/45 during gamete fusion currently makes it impossible to determine whether the efficacy of antibodies correlates with their effects on protein function. However, our structural characterisation of antibody epitopes, coupled with kinetic data and measurements of transmission-blocking activity, suggest that epitope accessibility is a key feature of highly potent antibodies. In particular, comparison of 85RF45.1 and 32F3 reveals that these two antibodies have overlapping epitopes on the C-terminal domain and have very similar affinities. The flexible nature of the 32F3 epitope, compared with the more rigid epitope for 85RF45.1 affects their kinetics of binding to soluble Pfs48/45 protein, with 85RF45.1 binding and dissociating more rapidly. However, unlike antigens involved in erythrocyte invasion^35^, Pfs48/45 is exposed for significant periods of time on the gamete surface, making it unlikely that fast association-rates are important for antibody efficacy. Instead, the most likely cause of differences in potency of 85RF45.1 and 32F3 is the degree to which their epitopes are exposed on the gametocyte surface. While 32F3 approaches from the side of the disc, 85RF45.1 binds on the more exposed, membrane-distal surface, allowing greater accessibility to its epitope in the context of the gametocyte surface. Indeed, transmission-blocking antibody 10D8 has lower efficacy than either 85RF45.1 and 32F3 and binds to an epitope in a less exposed position on the Pfs48/45 surface. However, exposure and accessibility does not provide the full story. Antibodies 3H6 and 9A6 bind to more exposed surfaces of Pfs48/45 than 10D8 and yet are not transmission blocking and do not bind to gametocytes, most likely due to occlusion by Pfs48/45 binding partners. Future studies are needed to reveal how its interactions with other binding partners modulates its activity and affect vaccine design decisions.

Finally, our studies of full-length Pfs48/45 have consequences for vaccine design. Many current approaches for Pfs48/45 have focused on the C-terminal domain, which is easier to produce than full-length Pfs48/45 and contains the epitopes for the most potent known transmission-blocking antibodies. However, the structure of Pfs48/45 shows that all three of its constituent domains will be exposed when membrane anchored. Our depletion studies also show that the majority of transmission-blocking antibodies raised through immunisation of mice with full-length Pfs48/45 bind to the N-terminal and central domains. There are still many uncertainties about the role of Pfs48/45 during gamete fusion, including when Pfs48/45 assembles into a complex with other gametocyte surface proteins or which epitopes are exposed when it is part of this complex. However, our current data suggest a vaccine design strategy which includes full-length Pfs48/45, to take advantage of epitopes for transmission-blocking antibodies that target the N-terminal and central domains, perhaps first generating versions of Pfs48/45 that are thermally stabilised to prevent dynamic movement. It also indicates the importance of correct presentation of Pfs48/45 during vaccination. Immunisation with soluble, or incorrectly oriented Pfs48/45, will induce antibodies that target epitopes poorly accessible on the gametocyte surface. In contrast, a vaccine particle which presents Pfs48/45 in an orientation consistent with that on the gametocyte surface is more likely to induce antibodies, like 85RF45.1, with potent transmission-blocking potential.

## Methods

### Pfs48/45 expression and purification

The sequences for all Pfs48/45 protein variants (PlasmoDB:PF3D7_1346700: Pfs48/45 full-length residues 27–427, Pfs48/45-D1 + 2 residues 27–288, Pfs48/45-D2 + 3 residues 159–428 and Pfs48/45-D3 residues 291–428) were codon optimised for expression in Drosophila melanogaster (GeneArt Life Technologies), with a Drosophila BiP signal peptide introduced at the N-terminus and the four amino acids EPEA (C-Tag) inserted at the C-terminus. All N-glycosylation sites were left intact. The sequence was subcloned into the *Drosophila* S2 expression vector pExpres2.2 (ExpreS2ion Biotechnologies). Polyclonal *Drosophila* S2 stable cell lines were generated by non-viral transfection into ExpreS2 *Drosophila* S2 cells (Expres2ions Biotechnologies) with selection via G418 resistance. Recombinant Pfs48/45-D1 + 2, Pfs48/45-D2 + 3 and Pfs48/45-D3 were purified by initially concentrating the cell culture supernatant using a Tangential Flow Filtration system with a Pellicon 3 Ultracel 3 kDa membrane (Merck Millipore, UK). Pfs48/45 full-length cell culture supernatant was concentrated as above but using a Pellicon 3 Ultracel 10 kDa membrane (Merck Millipore, UK). The concentrated supernatants were then loaded onto CaptureSelect™ C-tag affinity columns (Thermo Fisher Scientific) equilibrated in Tris-buffered saline and bound proteins were eluted with 20 mM Tris–HCl, 2 M MgCl_2_, pH 7.4. Protein containing fractions containing were then pooled and subjected to size-exclusion chromatography using a Superdex 200 16/600 PG column (Cytiva). Proteins were aliquoted and flash-frozen in liquid nitrogen for later use.

### Expression and purification of antibodies and scFvs

32F3, 85RF45.1, 10D8, 1F10, 3H6, 6A10 and 9A6 were expressed from hybridoma cell lines, purified using protein A/G and ficin cleaved to generate Fab fragments^12^. To produce scFv constructs, the sequence of 32F3 scFv was designed with the variable region of the heavy chain (from residue DVKLV to residue TLTVS) followed by an 18-residue linker (GGSSRSSSSGGGGSGGGG) and the variable region of light chain (from residue QIVLS to KLELK). The sequence of the 85RF45.1 scFv contains the variable region of the light chain (from residue QFVLS to residue KLTVT), followed by the 18-residue linker and the variable region of the heavy chain (from residue EVQLV to residue MVTVS). The sequence of 10D8 scFv contains the variable region of the light chain (from residue DIVMS to residue TKLEI), followed by the 18-residue linker and the variable region of the heavy chain (from residue EVMLV to residue GTSVT). Codon optimised synthetic genes (ThermoFisher) of 10D8 scFv, 32F3 scFv, and 85RF45.1 scFv were PCR amplified and subcloned into a pHLsec vector containing a C-terminal hexa-histidine tag. These were expressed in HEK293 cells. Cultures were harvested six days after transfection. The supernatant was filtered and incubated with Excel Ni Sepharose resin (GE Healthcare) at 4 °C for 30 min. The mixture was then applied onto a gravity flow column. The column was washed with 20 mM Tris pH 8.0, 150 mM NaCl, 10 mM imidazole and eluted in the same buffer also containing 250 mM imidazole. Elution fractions were collected and concentrated to run gel filtration with superdex 200 increase 10/300 (GE Healthcare) in 20 mM Tris pH 8.0, 150 mM NaCl.

### Crystallisation and structure determination

Complexes containing different combinations of full-length Pfs48/45, the C-terminal domain of Pfs48/45 (Pfs48/45-D3) and the central and C-terminal domains (Pfs48/45-D2 + 3), with one or more Fab fragment or scFv, were prepared by mixing different components in stoichiometric ratios. Complexes were purified by size exclusion chromatography on two Superdex Increase 200 10/300 columns (GE Healthcare) in series into 10 mM HEPES, 150 mM NaCl, pH 7.2. Peak fractions containing pure complex were then concentrated and used to set up vapour diffusion crystallisation trials in sitting drops by mixing 100 nl of protein complex with 100 nl of well solutions.

Crystals formed in the following conditions. For Pfs48/45-D3:32F3, initial crystals formed at a protein concentration of 11.1 mg/ml at 293 K in 0.04 M potassium dihydrogen phosphate, 16% (w/v) polyethylene glycol (PEG) 8000 and 20% (v/v) glycerol. Crystallisation conditions were further optimised by increasing the concentration of polyethylene glycol 8000 to 25% (w/v) and omitting potassium dihydrogen phosphate during crystallisation. For data collection, these crystals were transferred into well solution containing 30% (v/v) glycerol and flash-cooled in liquid nitrogen. For Pfs48/45-D2 + 3:10D8:32F3, crystals formed at a protein concentration of 10.5 mg/ml at 293 K in 10% (w/v) PEG monomethyl ether 5000, 0.1 M 2-(N-morpholino) ethanesulfonic acid pH 6.5 and 12% (v/v) 1-propanol. Initial crystals were further optimised by micro-seeding into the same condition. For data collection, crystals were transferred into well solution containing 25% (v/v) 2-methyl-2,4-pentanediol and flash-cooled in liquid nitrogen. For Pfs48/45-FL:10D8, the best crystals grew at a protein concentration of 10.7 mg/ml at 277 K in 10% (w/v) PEG 1000 and 10% (w/v) PEG 8000. For data collection, crystals were transferred into well solution containing 25% (v/v) 2-methyl-2,4-pentanediol and flash-cooled in liquid nitrogen. Crystals for Pfs48/45-FL:85RF45.1:10D8 formed at a protein concentration of 13.4 mg/ml at 277 K in 0.1 M Lithium sulfate, 0.1 M HEPES pH 7.0 and 20% w/v Polyvinylpyrrolidone. For data collection, crystals were transferred into well solution containing 30% (v/v) glycerol and flash-cooled in liquid nitrogen. For Pfs48/45FL:32F3 scFv, crystals formed at protein concentration of 12 mg/ml at 291 K in 0.1 M MES pH 6.0, 0.2 M CaCl_2_, 20% (w/v) PEG6000 and for data collection crystals were transferred into well solution containing 25% (v/v) glycerol.

Data were collected on the following beamlines. Data for Pfs48/45-D3:32F3 and for Pfs48/45-FL:10D8 were collected at IO4 (Diamond Light Source, UK); data for Pfs48/45-D2 + 3:10D8:32F3 were collected at IO4-1 (Diamond Light Source, UK); data for Pfs48/45-FL:85RF45.1:10D8 were collected at IO3 (Diamond Light Source, UK) and data for Pfs48/45-FL:32F3 scFv were collected at IO4 (Diamond Light Source, UK). The data for Pfs48/45-FL:85RF45.1:10D8, Pfs48/45-D3:32F3, Pfs48/45-D2 + 3:10D8:32F3 and Pfs48/45-FL:10D8 were processed with the CCP4i2 programme suite^36^ using the Xia2/DIALS v3.3 pipeline^37^. For Pfs48/45FL:85RF45.1:10D8, two datasets from the same crystal were merged together. Data for Pfs48/45-FL:32F3 scFv were processed with XDS. All structures were determined by molecular replacement using Phaser-MR v2.8.3^38^ with model building in Coot 0.9.4^39^ and refinement using phenix.refine^40^ and Buster v2.1^41^. All structure figures were prepared using PyMol v2.5.2 (Schroedinger LLC).

### Surface plasmon resonance analysis

Surface plasmon resonance experiments were carried out using a Biacore T200 instrument (GE Healthcare), to analyse binding of Pfs48/45 to 85RF45.1, 32F3 and 10D8. Pfs48/45 was buffer-exchanged into 20 mM HEPES pH 7.2, 300 mM NaCl, 0.05% Tween-20. Individual mAbs were immobilised on a CM5-chip (GE Healthcare) pre-coupled to Protein A/G (Thermo Fisher) and Pfs48/45 was injected over the chip surface at a flow rate of 30 µl/min, with 240 s association time and 600 s dissociation time. For all three antibodies, we injected a series of samples, forming two-fold dilution series, with a top concentration of 7.8 nM for 85RF45.1 and 125 nM for 32F3 and 10D8. After each injection, the chip surface was regenerated with 10 mM Glycine, pH 2.0 for 120 s at 10 µl/min, followed by a regeneration period of 180 s. Data were analysed using the BIAevaluation software 2.0.3 (GE Healthcare).

### Molecular dynamics simulations

To prepare structures for simulation, we first completed models for the missing regions in the Pfs48/45-FL:85RF45.1:10D8 and Pfs48/45-FL:32F3 scFv structures by grafting the model for the N-terminal domain from the Pfs48/45-FL:10D8 structure on the existing partial N-terminal domain models in the former two structures. Next, we modelled missing loops in each structure individually using MODELLER (v. 10.2)^42^. We allowed refinement only in the missing loops and generated 1000 decoys corresponding to residues 45–428 for each structure and picked the final models using the SOAP^43^ scoring function. We used OpenMM (v. 7.7)^44^ to run atomistic molecular dynamics simulations for each starting structure. To avoid non-physiological interactions due to truncation, we capped N- and C-termini using acetyl and N-methyl groups, respectively. Next, we protonated the models at a pH of 7.5, soaked them in truncated octahedral water boxes with a padding distance of 1 nm, and added NaCl to an ionic strength of 150 mM to neutralise charges. We parameterised the systems using the Amber14-SB force field^45^ and modelled water molecules using the TIP3P-FB model. Non-bonded interactions were calculated using the particle mesh Ewald^46^ method using a cut-off of distance of 0.9 nm, with an error tolerance of 0.0005. Water molecules and heavy atom-hydrogen bonds were rigidified using the SETTLE^47^ and SHAKE^48^ algorithms, respectively. We used hydrogen mass repartitioning^49^ to allow for 4 fs time steps. Simulations were run using the mixed-precision CUDA platform in OpenMM using the Middle Langevin Integrator and the Monte-Carlo Barostat. We equilibrated systems using a multi-step protocol: (i) energy minimisation over 10,000 steps, (ii) heating of the NVT ensembles from 100 K to 300 K over 200 ps, (iii) 200 ps simulation of the NPT ensembles at 300 K, (iii) cooling of the NVT ensembles from 300 K to 100 K over 200 ps, (iv) energy minimisation over 10,000 steps, (v) heating of the NVT ensembles from 100 K to 300 K over 200 ps, and (vi) 5 ns simulation of the NPT ensembles at 300 K. Following this, we ran production simulations of NPT ensembles for 500 ns. We used MDTraj (v. 1.9.6)^50^ for analyses of resulting trajectories.

### Small angle X-ray scattering analysis

Data for size exclusion chromatography-coupled small angle X-ray scattering (SEC-SAXS) of full-length Pfs48/45 were collected at the EMBL P12 BioSAXS beamline^51^ located at the PETRA III storage ring (DESY, Hamburg), at a wavelength of 1.24 Å using a Pilatus 2 M detector (Dectris, Baden-Daettwil, Switzerland) with a sample-detector distance of 3 m. For data collection, Pfs48/45 was concentrated to 3.25 mg/ml and injected over a Superdex Increase 3.2/300 column (GE Healthcare) equilibrated with 20 mM Hepes, 150 mM NaCl, pH 7.0. Frames were collected with an exposure time of 1 s. Data were processed with the CHROMIXS^52^ and ATSAS v3.0.3^53^ software suites, subtracting averaged buffer frames from averaged frames corresponding to the Pfs48/45 peak. The radius of gyration Rg was calculated using AutoRg in PRIMUS^54^, the distance distribution function P(r) and the maximum particle diameter Dmax were calculated using GNOM^55^. Volumetric representations were created by first calculating 20 ab initio bead models using DAMMIF^56^, followed by averaging of these models with DAMAVER^57^ and subsequent refinement against the original data with DAMMIN^58^. From the resulting model, an envelope was calculated using Situs, and models of Pfs48/45 were each fitted into this envelope using Chimera^59^. Figures were prepared with PyMol (Schroedinger LLC). Assessment of the fit of models derived from molecular dynamics to SAXS data was conducted using FoxS^31^. We we removed all N-methyl and acetyl caps as well as hydrogen atoms from the models and FoxS was used with the default settings.

### Negative stain electron microscopy and image processing

Each Fab was mixed with full-length Pfs48/45 and 85RF45.1 Fab at the ratio of 1:1:1 and incubated on ice for 30 min in 20 mM Tris pH 7.5, 150 mM NaCl. The mixture was then injected onto Superdex 200 Increase 10/300 column (Cytiva). Fractions corresponding to Pfs48/45 bound to two Fabs (around 150 kD) were collected and concentrated. Purified complexes were flash-frozen using liquid nitrogen and stored at −80 °C.

For negative staining, purified complexes of Pfs48/45:85RF45.1 Fab with one additional Fab, from 1F10 Fab, 3H6 Fab, 6A10 Fab, 9A6 Fab or 10D8 Fab were diluted to 10 µg/ml. Carbon support 200-mesh copper grids were glow-discharged at 15 mA for 20 s. 5 µl protein sample was applied onto the grids for 60 s and blotted. Then the grids were washed twice with 20 mM Tris pH 7.5, 150 mM NaCl buffer and stained twice with 2% uranyl acetate for 60 s and blotted.

100–120 micrographs were collected for each sample on Talos F200c transmission electron microscope with a Ceta 16 M CMOS camera (Thermo Scientific). Nominal magnification was ×45,000 for the 1F10 dataset (pixel size 2.30 Å), and ×57,000 for the other datasets (pixel size 1.8 Å). The defocus was set at −5 µm for the 1F10 dataset, and at −1.5 µm for other datasets.

Data were first processed using SIMPLE v3.0^60^. The micrographs had their contrast transfer functions estimated, and particles were automatically picked based on 2D references generated from manually picked particles. 10,000 to 30,000 particles were selected after two rounds of 2D classification and selected to build initial 3D models using SIMPLE. The particles were then exported to RELION v3.1^61,62^ followed by a round of 2D classification. Around 10k particles were selected for 3D classification using the initial 3D model generated from SIMPLE. 3D refinement was then conducted on one of the classes from the 3D classification.

### Immunisation of mice

Animal experiments and procedures were performed according to the UK Animals (Scientific Procedures) Act Project License (PA7D20B85) and approved by the Oxford University Local Ethical Review Body. Six- to eight-week-old female CD-1 mice (Envigo RMS Inc.), housed in specific-pathogen-free environments, were vaccinated with full-length Pfs48/45, Pfs48/45-D1 + 2, or Pfs25, into each leg via the intramuscular route, using a prime-boost regime with a 3-week interval, with blood being collected 3 weeks after the final dose. Immunisations were formulated in adjuvant prior to vaccination by mixing Alhydrogel® adjuvant 2% (Invivogen; 85 μg per dose) with antigen in TBS, and incubated at RT for 1 h before injection. Sera were obtained from whole blood by leaving samples overnight at 4 °C to clot, followed by 10 min centrifugation at 16,000 × *g* in a benchtop centrifuge at room temperature.

### Enzyme-linked immunosorbent assays

For endpoint ELISAs, Nunc-Immuno Maxisorp 96-well plates (Thermo Scientific) coated with 2 μg/ml of antigen in PBS (Sigma) overnight at 4 °C. Plates were washed with PBS-Tween (0.05%) and blocked with 10% Casein Block (Thermo Scientific). Diluted samples for endpoint ELISAs were added to the top row of the plate in duplicate, and used to generate a threefold serial dilution. Plates were incubated for 2 h at room temperature and then washed as before. Goat anti-mouse whole IgG conjugated to alkaline phosphatase (Sigma) was added for 1 h at room temperature. Following a final wash, plates were developed by adding *p*-nitrophenylphosphate (Sigma) at 1 mg/mL in diethanolamine buffer (Sigma) and OD_405nm_ was read on an ELx808 microtitre plate reader (Biotek). The endpoint titre is defined as the x-axis intercept of the dilution curve at an absorbance value (±three standard deviations) greater than the OD for a serum sample from a naive mouse. If a 1:250 dilution of a serum sample did not develop a signal above background, the IgG titre in this sample was considered to be 0. Sera from a pool of mice with a strong anti Pfs48/45-D1 + 2 response were included on each plate as an internal control, and all plates developed until this control reached an endpoint titre of 1,350,000–1,650,000. The sera of mice immunised with Pfs25 was included as a negative control.

To validate the depletion of Pfs48/45-D3 specificity from purified IgG, ELISAs were conducted as above with Pfs48/45-D3 being used as the coating antigen and IgG being serially diluted threefold down the plate from an initial concentration of 0.01 mg/mL. Raw OD405_nm_ is presented. Pfs25-specific IgG was included as a negative control.

### Standard membrane feeding assays

The ability of vaccine-induced antibodies to block the development of *P. falciparum* strain NF54 was evaluated by SMFA as previously described^63^. The percentage of mature stage V gametocytes was adjusted to 0.15 ± 0.05% and the male-female ratio was stable (almost always 1 male: 2–3 female). These were mixed with purified IgG at the concentrations (diluted in PBS) shown in the figures and then fed to 4–6 days old starved female *Anopheles stephensi* (SDA 500) via a parafilm membrane. The mosquitoes were maintained at 26 °C and 80% relative humidity. After 8 days, midguts from 20 mosquitoes per group were dissected, oocysts counted, and the number of infected mosquitoes recorded. Percent reduction in infection intensity was calculated relative to the respective control IgG tested (Normal mouse IgG) in the same assay. The monoclonal antibody 4B7, specific for Pfs25 was included as a positive control at 94 μg/ml.

### Statistical analysis

Comparison of endpoint ELISA data between sera of the two groups of mice was performed by a Mann–Whitney test. The 95% confidence intervals (95% CI), and *p*-values of SMFA results were calculated using a zero-inflated negative binomial random effects model described previously^64^.

### Reporting summary

Further information on research design is available in the Nature Research Reporting Summary linked to this article.

## Supplementary information

Supplementary Information

Reporting Summary

## Data Availability

Coordinates and structure factors derived in this study have been deposited in the Protein Data Bank under accession codes 7ZWF, 7ZWI, 7ZWM, 7ZXF and 7ZXG. Source data for all graphs generated in this study are provided in “source data.xlsx”. Source data are provided with this paper.

## Source data

Source Data
