# Supplementary material for: Structure of the malaria vaccine candidate Pfs48/45 and its recognition by transmission blocking antibodies
Source: Nat Commun. 2022 Sep 24;13:5603. doi: 10.1038/s41467-022-33379-6 (PMC9509318; doi:10.1038/s41467-022-33379-6)
Supplement: Supplementary file 1 — Supplementary Information [file 41467_2022_33379_MOESM1_ESM.pdf]

Supplementary Information

Structure of the malaria vaccine candidate Pfs48/45 and its recognition by transmission blocking antibodies

Supplementary Data Figures and Tables

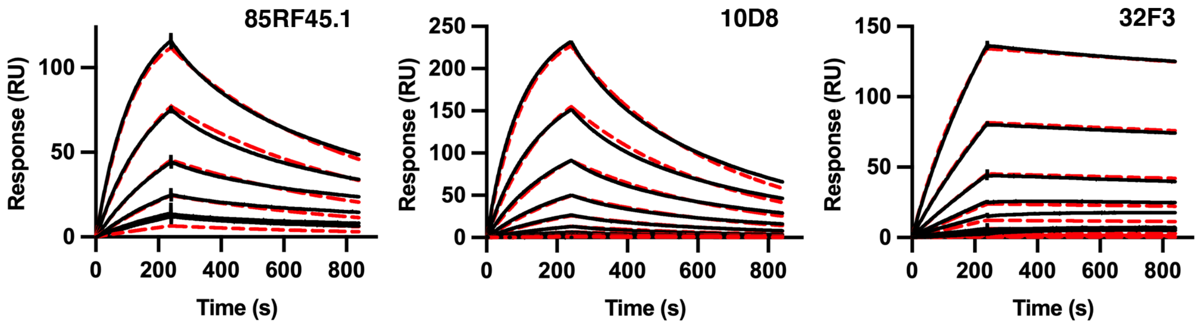

	k_a (1/Ms)	k_d (1/s)	K_D (M)	chi^2
10D8	7.55×10^4	0.002702	3.58×10^{-8}	5.23
32F3	2.94×10^6	1.21×10^{-4}	4.10×10^{-9}	8.52
85RF45.1	1.38×10^6	0.001862	1.34×10^{-9}	6.58

Supplementary Figure 1: surface plasmon resonance analysis

The top panel shows surface plasmon resonance traces for binding to immobilised Pfs48/45 for antibodies 85RF45.1 (a two-fold dilution series from a top concentration of 7.8nM), 10D8 and 32F3 (each two-fold concentrations series from top concentrations of 125nM). The red dotted lines show the fitting to a one-to-one binding model. The lower panel shows the binding kinetics for these three sets of data based on the one-to-one fitting.

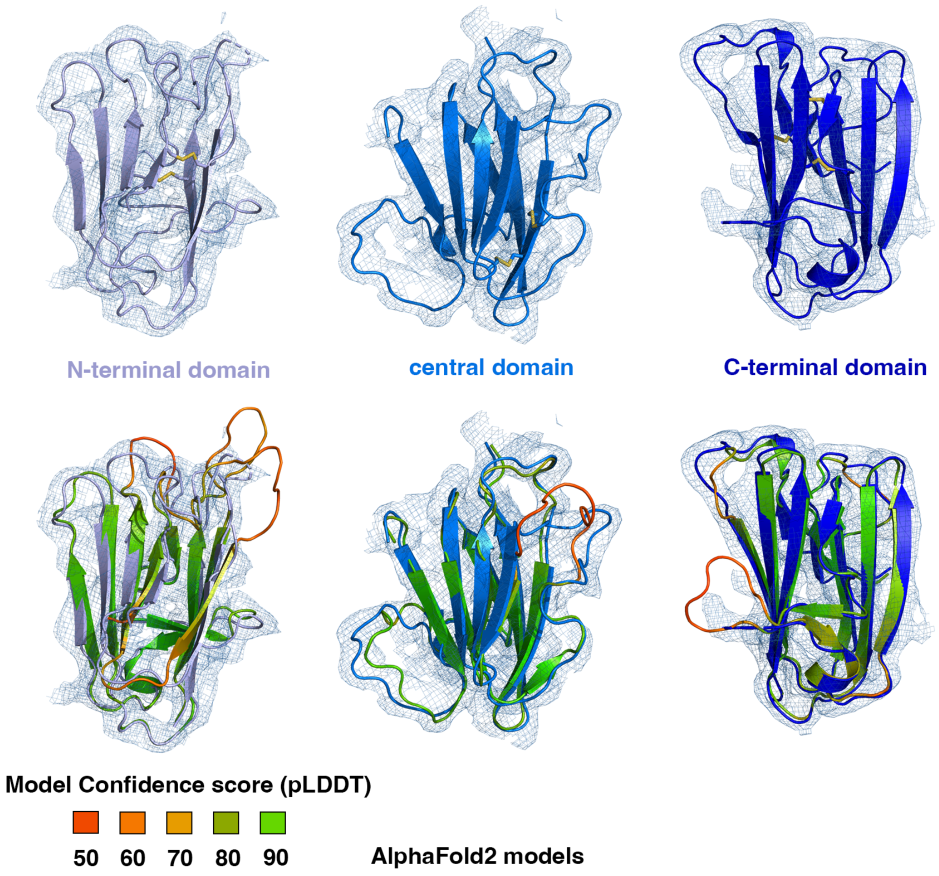

Supplementary Figure 2: the domains of Pfs48/45 determined by x-ray crystallography compared with AlphaFold2 models

The upper panels show the N-terminal, central and C-terminal domains of Pfs48/45. Disulphide bonds are shown as sticks, with sulphur residues coloured yellow. These are taken from the structure of full-length Pfs48/45 in complex with 10D8, and the equivalent map is shown at a contour level of 1.0 in blue mesh. The lower panel shows the same three domains aligned with the equivalent AlphaFold2 model. AlphaFold models are shown in the colour scheme indicated at the bottom of the figure, with green used for regions of highest confidence and red for lowest confidence.

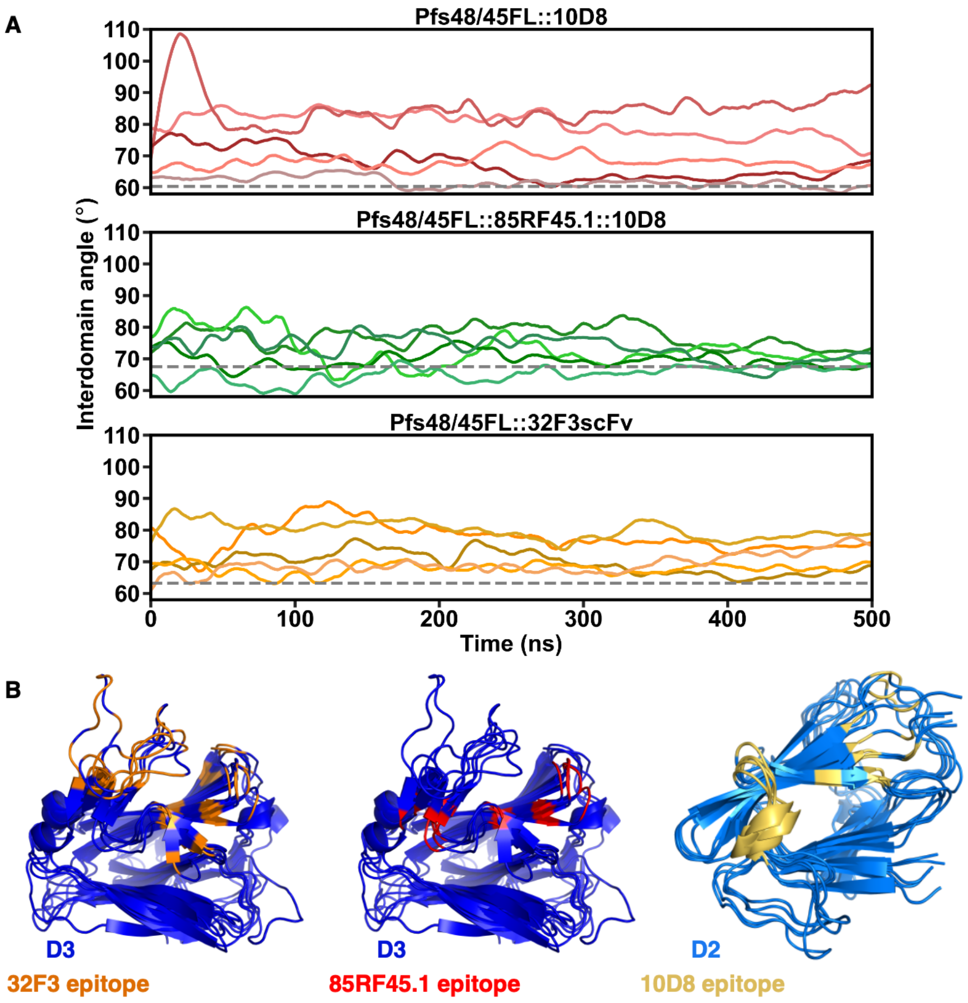

Supplementary Figure 3: molecular dynamics simulations

A. Graphs showing the observed interdomain angles in full-length Pfs48/45 during atomistic molecular dynamics simulations. Three independent sets of simulations were run, using full-length Pfs48/45 taken from the crystal structure of the complex indicated above each graph. The interdomain angle is that between the line linking the centres of mass of the N-terminal and central domains and that linking the centres of mass of the central and C-terminal domains. The five lines on each graph show five independent simulations and were smoothened using a Savistky-Golay filter. **B.** A representation of the degree of flexibility of the epitopes for 32F3 (left), 85RF45.1 (centre) and 10D8 (right). In each case, the structure of Pfs48/45 is shown in blue with the residues which contact the antibody in orange, red or yellow. Five structures are shown, from the simulations shown in **A**, to indicate the degree of epitope flexibility.

A

Protein	Rg_{exp} (nm)	D_{max} (nm)	V_{porod} (nm^3)	Mr_{exp} (kDa)	Mr_{app} (kDa)
Pfs48/45FL	2.69	8.15	86.91	46.00	59.00

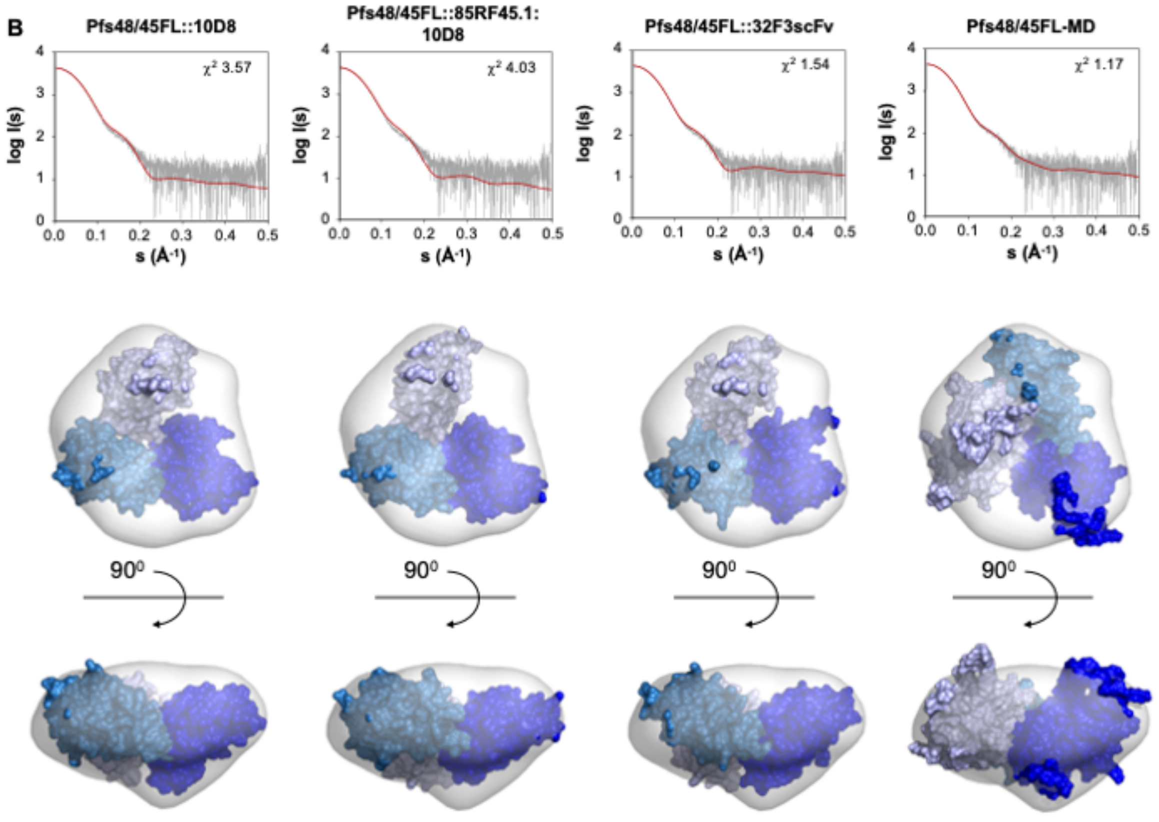

Supplementary Figure 4: small angle x-ray scattering

A. Parameters derived from solution scattering data. Rg_{exp} is the radius of gyration, D_{max} the maximum particle diameter, V_{Porod} is the Porod volume. Mr_{app} is the molecular mass calculated from the excluded volume in the final volumetric representation, and Mr_{exp} is the expected molecular mass. **B.** Fit between the SEC-SAXS data and the three structures of Pfs48/45 constructed based on the crystal structures of Pfs48/45 bound to different antibody combinations and the structure from MD simulations conducted on Pfs48/45 that shows the lowest χ^2 when fitted to the SEC-SAXS data (Pfs48/45FL-MD). The red line shows the scattering curve calculated from the model and the grey line shows scattering data. Shown below are the fits of these structures to the envelope calculated from the SEC-SAXS data, docked using Chimera.

1F10:: Pfs48/45:: 85RF45.1

3H6:: Pfs48/45:: 85RF45.1

6A10:: Pfs48/45:: 85RF45.1

9A6:: Pfs48/45:: 85RF45.1

10D8:: Pfs48/45:: 85RF45.1

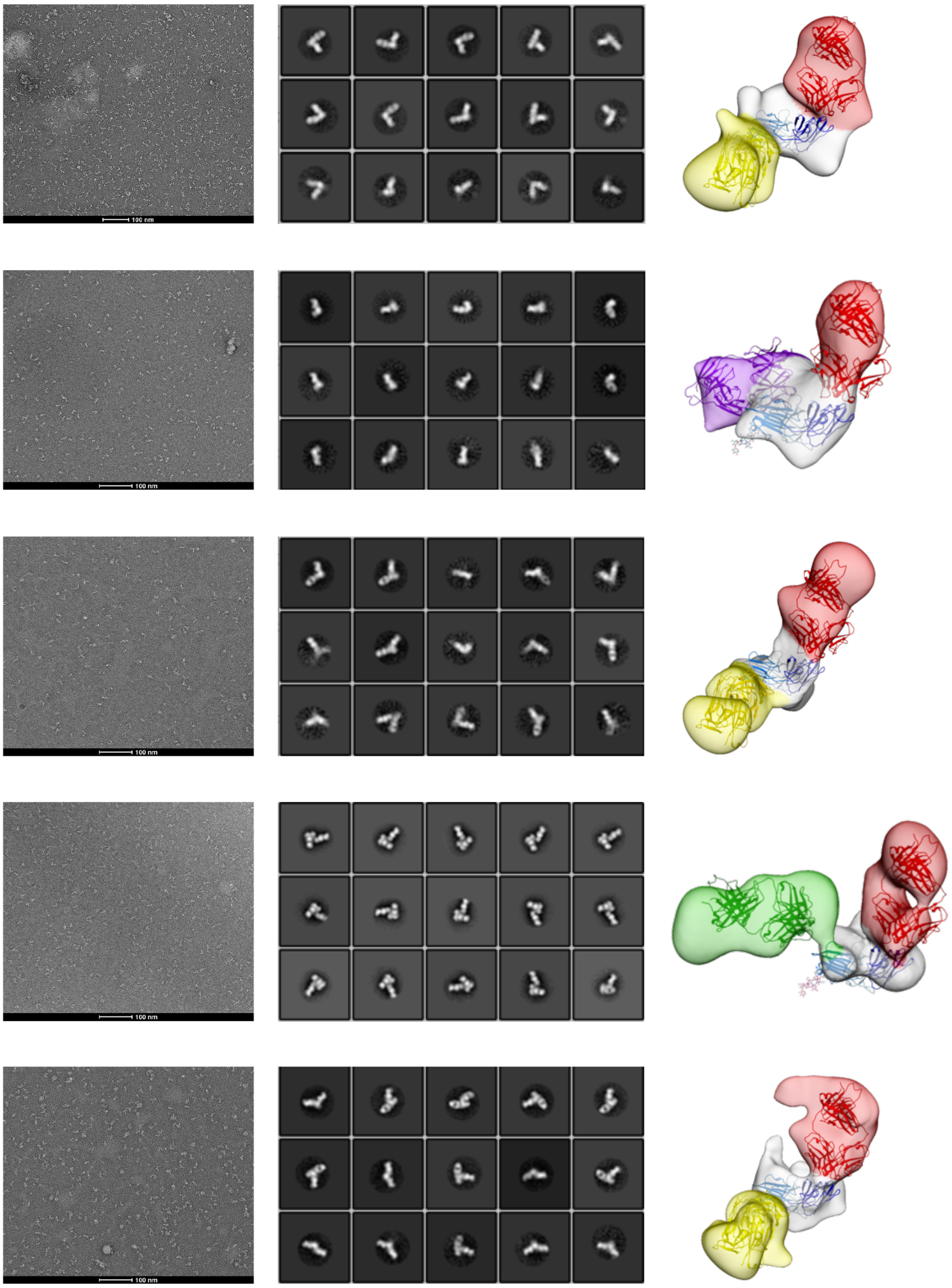

Supplementary Figure 5: negative stain electron microscopy

Electron microscopy imaging of complexes containing Pfs48/45 (white), the Fab of 85RF45.1 (red) and one additional antibody Fab, as indicated to the left-hand side of each row.. Each row shows a representative micrograph (n>100 as described in Supplementary Table 4, left), two-dimensional class averages (centre) and a low-resolution reconstruction (right).

Supplementary Table 1: crystallographic statistics for structures with incomplete Pfs48/45 constructs

**Data collection**	**Pfs48/45-6C:32F3**	**Pfs48/45-D2+3:10D8:32F3**
Space group	$P12_11$	$P2_12_12_1$
Cell dimensions:		
a, b, c	78.66, 78.66, 110.62	108.58, 158.45, 186.16
α, β, γ	90°, 99.5°, 90°	90°, 90°, 90°
Resolution	77.57 – 1.90 Å (1.93 – 1.90Å)	108.58 – 3.69 Å (3.75 – 3.69Å)
Total observations	781771 (36476)	230227 (11072)
Total unique	116014 (5704)	35355 (1713)
R_{pim}	0.037 (0.282)	0.065 (0.338)
$CC_{1/2}$	0.999 (0.780)	0.971 (0.438)
I/σ(I)	11.6 (2.2)	7.2 (1.8)
Completeness (%)	99.9 (97.8)	100.0 (98.0)
Multiplicity	6.7 (6.4)	6.5 (6.5)
Wilson B factor ($Å^2$)	27	119
**Refinement**		
Number of reflections	115991	35298
R_{work} / R_{free}	0.189 / 0.217	25.5 / 28.0
B factors ($Å^2$)		
Average	42	161
Pfs48/45-D2	N/A	(A) 169 (F) 157
Pfs48/45-D3	(A) 34 (B) 32	(A) 138 (F) 145
32F3 heavy chain	(B) 41 (E) 44	(B) 144 (G) 145
32F3 light chain	(C) 48 (F) 48	(C) 153 (H) 136
10D8 heavy chain	N/A	(D) 157 (I) 189
10D8 light chain	N/A	(E) 159 (J) 220
Number of residues:		
Amino acid residues	1123	2179
Sugars	3	10
Waters	963	0
RMSZ deviations		
Bond lengths	0.009	0.004
Bond angles	1.02	0.67
Ramachandran plot		
Favoured	98.5 %	94.8 %
Allowed	1.5 %	5.2 %
Outliers	0 %	0 %

Supplementary Table 2: crystallographic statistics for structures with complete Pfs48/45 constructs

**Data collection**	**Pfs48/45-FL:10D8**	**Pfs48/45-FL:10D8: 85RF45.1**	**Pfs48/45-FL: 32F3 scFv**
Space group	$P2_122_1$	$P4_12_12$	$C222_1$
Cell dimensions:			
a, b, c	81.77, 125.80, 216.60	156.88, 156.80, 148.76	81.83, 126.12, 146.79
α, β, γ	90°, 90°, 90°	90°, 90°, 90°	90°, 90°, 90°
Resolution	76.27 – 4.20 Å (4.70 – 4.20Å)	108.00 – 3.72 Å (4.06 – 3.72Å)	73.39 – 2.13 Å (2.17 – 2.13Å)
Total observations	109594 (31747)	456518 (56247)	586552 (28745)
Total unique	16968 (4732)	17015 (1893)	42648 (2277)
R_{pim}	0.088 (0.668)	0.027 (0.875)	0.037 (2.26)
$CC_{1/2}$	0.998 (0.543)	0.999 (0.377)	0.999 (0.384)
I/σ(I)	7.5 (1.6)	15.7 (1.2)	11.2 (0.4)
Completeness (%)	99.9 (100.0)	94.9 (74.3)	99.8 (99.4)
Multiplicity	6.5 (6.7)	26.8 (29.7)	13.8 (14.2)
Wilson B factor ($Å^2$)	166	191	59
**Refinement**			
Number of reflections	16933	16936	40794
R_{work} / R_{free}	25.9 / 27.6	28.1 / 30.2	27.6 / 28.6
B factor ($Å^2$)			
Average	212	215	98
Pfs48/45-D1	(A) 265 (D) 270	249	178
Pfs48/45-D2	(A) 199 (D) 195	212	130
Pfs48/45-D3	(A) 213 (D) 191	190	86
32F3scFv	N/A	N/A	83
85RF45.1 heavy	N/A	202	N/A
85RF45.1 light	N/A	22	N/A
10D8 heavy chain	(B) 180 (E) 204	210	N/A
10D8 light chain	(C) 194 (F) 231	230	N/A
Number of residues:			
Amino acid residues	1574	1190	497
Sugars	15	8	2
Waters	0	0	94
RMSZ deviations			
Bond lengths	0.003	0.003	0.008
Bond angles	0.661	0.593	0.99
Ramachandran plot			
Favoured	91.9 %	92.8 %	92.8 %
Allowed	8.1 %	7.2 %	7.2 %
Outliers	0 %	0 %	0 %

Supplementary Table 3: Interactions between Pfs48/45 and antibodies 32F3 and 10D8

Pfs48/45-6C		32F3 Fab		
Residue	Group	Chain/Residue	Group	Type of interaction
Ser322	Mainchain (O)	Heavy/ Arg98	Sidechain	Hydrogen bond
Ser322	Mainchain (O)	Heavy/ Tyr32	Sidechain	Hydrogen bond
Ser322	Sidechain	Heavy/ Tyr109	Sidechain	Hydrogen bond
His324	Sidechain	Heavy/ Tyr32	Sidechain	Hydrogen bond
Tyr357	Sidechain	Heavy/ Tyr101	Mainchain (O)	Hydrogen bond
Glu360	Sidechain	Heavy/ Tyr33	Sidechain	Hydrogen bond
Glu363	Sidechain	Heavy/ Ser57	Sidechain	Hydrogen bond
Thr409	Sidechain	Heavy/ Tyr101	Sidechain	Hydrogen bond
Asp347	Sidechain	Light/ Ser52	Sidechain	Hydrogen bond
Leu364	Mainchain (O)	Light/ Asn93	Sidechain	Hydrogen bond
Glu365	Sidechain	Light/ Asn93	Sidechain	Hydrogen bond
Asn368	Sidechain	Light/ Ser91	Sidechain, mainchain	Hydrogen bond
Ile369	Mainchain (N)	Light/ Thr30	Sidechain	Hydrogen bond
Lys413	Sidechain	Light/ Gln48	Sidechain	Hydrogen bond
Lys413	Sidechain	Light/ Ser52	Sidechain	Hydrogen bond
Lys413	Sidechain	Light/ Leu53	Sidechain	Hydrogen bond
Asp415	Mainchain (O)	Light/ Ser55	Sidechain	Hydrogen bond
Ile349, Ile411	Sidechain	Heavy/ Leu104	Sidechain	Hydrophobic
Pro350, Ile369	Sidechain	Heavy/ Leu104<br>Light/ Tyr31	Sidechain	Hydrophobic
Tyr357, Tyr420	Sidechain	Heavy/ Tyr101<br>Heavy/ Leu104<br>Heavy/ Tyr105	Sidechain	Hydrophobic
Pro366	Sidechain	Light/ Trp90	Sidechain	Hydrophobic

Pfs48/45-FL		10D8Fab		
Residue	Group	Chain/Residue	Group	Type of interaction
Ser206	Mainchain (O)	Light/ Asn34	Sidechain	Hydrogen bond
Asn207	Sidechain	Heavy/ Asp104	Mainchain (O)	Hydrogen bond
Phe208	Mainchain (O)	Heavy/ Arg105	Sidechain	Hydrogen bond
Glu214	Sidechain	Heavy/ His59	Sidechain	Hydrogen bond
Glu216	Sidechain	Heavy/ Ser52	Sidechain	Hydrogen bond
Glu216	Sidechain	Heavy/ Ser54	Mainchain (N)	Hydrogen bond
Glu216	Sidechain	Heavy/ Ser56	Sidechain, mainchain	Hydrogen bond
Lys287	Sidechain	Heavy/ Ser56	Mainchain (O)	Hydrogen bond
Glu205	Sidechain	Light/ Ser33	sidechain	Hydrogen bond
Phe208	Mainchain (O)	Light/ Tyr31	Sidechain	Hydrogen bond
Phe208	Mainchain (O)	Heavy/ Arg105	Sidechain	Hydrogen bond
Val209	Sidechain	Heavy/ Tyr57<br>Heavy/ Leu107<br>Light/ Tyr100	Sidechain	Hydrophobic

Supplementary Table 4: Negative stain electron microscopy data collection information

	**1F10: Pfs48/45: 85RF45.1**	**3H6: Pfs48/45: 85RF45.1**	**6A10: Pfs48/45: 85RF45.1**	**9A6: Pfs48/45: 85RF45.1**	**10D8: Pfs48/45: 85RF45.1**
Microscope	Talos F200c	Talos F200c	Talos F200c	Talos F200c	Talos F200c
Voltage (kV)	200	200	200	200	200
Magnification	45,000	57,000	57,000	57,000	57,000
Pixel size (Å)	2.3	1.8	1.8	1.8	1.8
Defocus (µm)	-5	-1.5	-1.5	-1.5	-1.5
Number of micrographs	101	116	120	111	101
Number of picked particles	95,717	51,993	94,752	67,240	57,853
Particles after 2D classification	27,759	7,586	4,893	13,780	7,988
Particles in the final map	10,137	1,815	3,319	2,925	4,664
