# Supplementary material for: Structure of the malaria vaccine candidate Pfs48/45 and its recognition by transmission blocking antibodies
Source: Nat Commun. 2022 Sep 24;13:5603. doi: 10.1038/s41467-022-33379-6 (PMC9509318; doi:10.1038/s41467-022-33379-6)
Supplement: Supplementary file 2 — Reporting Summary [file 41467_2022_33379_MOESM2_ESM.pdf]

nature portfolio

Corresponding author(s): Sumi Biswas and Matthew Higgins

Last updated by author(s): 07/09/2022

Reporting Summary

Nature Portfolio wishes to improve the reproducibility of the work that we publish. This form provides structure for consistency and transparency in reporting. For further information on Nature Portfolio policies, see our Editorial Policies and the Editorial Policy Checklist.

Statistics

For all statistical analyses, confirm that the following items are present in the figure legend, table legend, main text, or Methods section.

n/a	Confirmed	
☐	☒	The exact sample size (*n*) for each experimental group/condition, given as a discrete number and unit of measurement
☐	☒	A statement on whether measurements were taken from distinct samples or whether the same sample was measured repeatedly
☐	☒	The statistical test(s) used AND whether they are one- or two-sided<br>*Only common tests should be described solely by name; describe more complex techniques in the Methods section.*
☒	☐	A description of all covariates tested
☒	☐	A description of any assumptions or corrections, such as tests of normality and adjustment for multiple comparisons
☐	☒	A full description of the statistical parameters including central tendency (e.g. means) or other basic estimates (e.g. regression coefficient) AND variation (e.g. standard deviation) or associated estimates of uncertainty (e.g. confidence intervals)
☐	☒	For null hypothesis testing, the test statistic (e.g. F, t, r) with confidence intervals, effect sizes, degrees of freedom and P value noted<br>*Give P values as exact values whenever suitable.*
☒	☐	For Bayesian analysis, information on the choice of priors and Markov chain Monte Carlo settings
☒	☐	For hierarchical and complex designs, identification of the appropriate level for tests and full reporting of outcomes
☒	☐	Estimates of effect sizes (e.g. Cohen's *d*, Pearson's *r*), indicating how they were calculated

Our web collection on statistics for biologists contains articles on many of the points above.

Software and code

Policy information about availability of computer code

Data collection	Data collection and processing software used are described in the methods section and are commercially available or openly accessible.
Data analysis	Data analysis was performed as described in the methods section using commercially available or openly accessible software. Software used for crystallographic data processing and structure determination is the standard in the field and is all freely available to academic users. BIAevaluate v2.0.3 was used for SPR analysis. DIALS v3.3, CCP4i2 v1.0.2, PHASER v2.8.3, BUSTER v2.1 and Coot v0.9.4, were used for crystallography analysis. MD used MODELLER v10.2 and OpenMM v7.7. ATSAS v3.0.3 and DENSS v1.6 were used for SAXS analysis. SIMPLE v3.0 and RELION v3.1 were used for negative stain EM analysis. Software used for crystallographic data processing and structure determination and SAXS analysis is the standard in the field and is all freely available to academic users.GraphPad Prism version 7 was used to generate graphs, PyMOL 2.5.2 for structure visualisation.

For manuscripts utilizing custom algorithms or software that are central to the research but not yet described in published literature, software must be made available to editors and reviewers. We strongly encourage code deposition in a community repository (e.g. GitHub). See the Nature Portfolio guidelines for submitting code & software for further information.

Data

Policy information about availability of data

All manuscripts must include a data availability statement. This statement should provide the following information, where applicable:

- Accession codes, unique identifiers, or web links for publicly available datasets
- A description of any restrictions on data availability
- For clinical datasets or third party data, please ensure that the statement adheres to our policy

Coordinates and structure factors derived in this study have been deposited in the Protein Data Bank under accession codes 7ZWF, 7ZWI, 7ZWM, 7ZXF and 7ZXG. Source data for all graphs generated in this study are provided in “source data.xlsx”.

Database datasets used in the manuscript are AlphaFold model entry https://alphafold.ebi.ac.uk/entry/V5NTZ4 and PDB code 6H5N.

Human research participants

Policy information about studies involving human research participants and Sex and Gender in Research.

Reporting on sex and gender	N/A
Population characteristics	N/A
Recruitment	N/A
Ethics oversight	N/A

Note that full information on the approval of the study protocol must also be provided in the manuscript.

Field-specific reporting

Please select the one below that is the best fit for your research. If you are not sure, read the appropriate sections before making your selection.

☒ Life sciences ☐ Behavioural & social sciences ☐ Ecological, evolutionary & environmental sciences

For a reference copy of the document with all sections, see nature.com/documents/nr-reporting-summary-flat.pdf

Life sciences study design

All studies must disclose on these points even when the disclosure is negative.

Sample size	For SMFA experiments, mosquito group size was chosen based on previous studies (63). For immunisation studies, no sample size calculation was performed and group sizes were chosen as to minimise animal use whilst still provide sufficient sample size to see significant differences based on previous experiments.
Data exclusions	No data were excluded from analysis.
Replication	All SPR experiments were repeated three times and gave equivalent outcomes to the data shown.
Randomization	No randomisation was conducted as no decisions about inclusion or exclusion of data were taken and experiments were designed such that single parameters were varied during the experiment. All data was included in analysis.
Blinding	No blinding was conducted as no subjective decisions about data inclusion were involved in data collection.

Reporting for specific materials, systems and methods

We require information from authors about some types of materials, experimental systems and methods used in many studies. Here, indicate whether each material, system or method listed is relevant to your study. If you are not sure if a list item applies to your research, read the appropriate section before selecting a response.

Materials & experimental systems

n/a	Involved in the study	
☐	☒	Antibodies
☐	☒	Eukaryotic cell lines
☒	☐	Palaeontology and archaeology
☐	☒	Animals and other organisms
☒	☐	Clinical data
☒	☐	Dual use research of concern

Methods

n/a	Involved in the study	
☒	☐	ChIP-seq
☒	☐	Flow cytometry
☒	☐	MRI-based neuroimaging

Antibodies

Antibodies used

Monoclonal antibodies 85RF45.1, 32F3, 10D8, 3H6, 9A6, 1F10, 4B7 and 6A10 are not commercially available. All were produced and validated as described in publications which are cited in the manuscript and were used at concentrates as described in the text. 4B7 was used at a concentration of 94µg/ml and was made using the murine hybridoma MRA-315 obtained from BEI resources, NIAID, NIH (Batch# GCN-L03p70). Pfs48/45 specific ELISAs used Goat anti-mouse whole IgG conjugated to alkaline phosphatase (Sigma A3562 Lot# SLCB8722) was added for 1 h at room temperature. Polyclonal sera was raised through immunisation of mice and was assessed for efficacy as described in the text.

Validation

All antibodies were previously validated through Western blotting and gametocyte staining in previous publications, as cited in the text.

Eukaryotic cell lines

Policy information about cell lines and Sex and Gender in Research

Cell line source(s)

Drosophila S2 cells were purchased from Expres2ions Biosciences for protein production and HEK293 cells were used to produce antibodies and antibody fragments. No cell-based experiments are included in this study and these cells were inly used to produce protein.

Authentication

No authentication was conducted as cells were only used to produce protein for analysis and the protein was validated instead of the cells.

Mycoplasma contamination

Not tested

Commonly misidentified lines
(See ICLAC register)

None were used.

Animals and other research organisms

Policy information about studies involving animals; ARRIVE guidelines recommended for reporting animal research, and Sex and Gender in Research

Laboratory animals

Six to eight weeks old female CD-1 mice were used for antibody production. These were kept in pathogen free housing with the temperature set at 21C +/- 10%, humidity 55% +/- 10% and 13 hours light/11 hours dark cycle.
Mosquitoes used for transmission-blocking assays were 4–6 days old female Anopheles stephensi (SDA 500).

Wild animals

None

Reporting on sex

The mice used were female

Field-collected samples

None

Ethics oversight

Animal experiments and procedures were performed according to the UK Animals (Scientific Procedures) Act Project License (PA7D20B85) and approved by the Oxford University Local Ethical Review Body.

Note that full information on the approval of the study protocol must also be provided in the manuscript.
